# Different Oxytocin Responses to Acute Methamphetamine Treatment in Juvenile Female Rats Perinatally Exposed to Stress and/or Methamphetamine Administration

**DOI:** 10.3389/fphys.2019.00305

**Published:** 2019-03-28

**Authors:** Anna Holubová, Silvester Poništ, Jana Jurčovičová, Romana Šlamberová

**Affiliations:** Department of Physiology, Third Faculty of Medicine, Charles University, Prague, Czechia

**Keywords:** oxytocin, methamphetamine, postnatal stress, prenatal stress, maternal separation

## Abstract

Methamphetamine (MA) is an addictive psychostimulant, often abused by drug-addicted women during pregnancy. The offspring of drug-addicted mothers are often exposed to perinatal stressors. The present study examines the effect of perinatal stressors and drug exposure on plasma oxytocin (OXY) levels in female progeny. Rat mothers were divided into three groups according to drug treatment during pregnancy: intact controls (C); saline (SA, s.c., 1 ml/kg); and MA (s.c., 5 mg/kg). Litters were divided into four groups according to postnatal stressors lasting from PD1 to 21: non-stressed controls (N); maternal separation (S); maternal cold-water stress (W); and maternal separation plus cold-water stress (SW). On postnatal day 30, acute MA or SA was administrated 1 h before the rats were sacrificed. Trunk blood was collected and plasma OXY was measured by specific ELISA after extraction. Our results showed that acute MA administration significantly increases plasma OXY levels in juvenile female rats; this effect was observed in prenatally intact rats only. Prenatal exposure of rats to mild stressor of daily SA injection prevented both acute MA-induced OXY stimulation and also stress-induced OXY inhibition. Although postnatal MA and stress exposure exert opposite effects on OXY release in juvenile rats, our data point out the modulatory role of prenatal mild stress in OXY response to postnatal stressors or MA treatment.

## Introduction

Exposure to stress early in life may be associated with impairment of neurological and endocrine development that can contribute to various long-lasting pathologies (Heim and Nemeroff, 2001; Marais et al., 2008; Lajud et al., 2012; Holubová et al., 2018). Periodic neonatal stress may stimulate the hypothalamic-pituitary-adrenal (HPA) axis response in pups even during the protective hypo-responsive period during the first two postnatal weeks (Sapolsky and Meaney, 1986; Lajud et al., 2012; Holubová et al., 2016). Besides HPA activation, various types of stressors also activate the hypothalamo-neurohypophyseal (HNP) tract resulting in release of oxytocin (OXY) directly into blood (Benarroch, 2013; Babb et al., 2014; Neumann and Slattery, 2016). OXY receptors are distributed throughout the brain, including regions that are involved in the stress response and emotional processing such as amygdala, hypothalamus, hippocampus, and mesocorticolimbic dopaminergic system, which is involved in the reward system (Baskerville and Douglas, 2010; Stoop, 2012). Thus, OXY, also called the “anti-stress hormone,” can significantly affect social behavior, including the mother-infant relationship and anxiety (Fahrbach et al., 1985; Insel, 2010; Labuschagne et al., 2010).

OXY system develops throughout gestation and postnatal period with the most critical early postnatal and adolescent periods (Baracz et al., 2018). Preclinical studies in prepubertal females found a significant increase in plasma OXY level (Babb et al., 2014; Minhas et al., 2016) as well as an increase in the size of the supraoptic (SON) and paraventricular nuclei (PVN) after acute restraint stress (Minhas et al., 2016). However, the response of oxytocin in juvenile rats exposed to chronic perinatal stressors has not been studied yet. Studies focusing on changes in OXY levels after stress exposure in adults are inconsistent. Some studies have found lower OXY concentrations in the cerebrospinal fluid of adult women (Heim et al., 2009), non-human primates (Winslow et al., 2003), and rodents (Champagne, 2008) associated with adverse early life experience. Moreover, a decrease in maternal care and nursing was associated with a decrease in hypothalamic OXY gene expression (Murgatroyd and Nephew, 2013). It should be noted that the relationship between central and peripheral OXY is not unequivocal. There are studies suggesting that the two oxytocinergic systems are independent of each other (Kagerbauer et al., 2013). However, some preclinical and clinical studies have found that the changes in peripheral plasma OXY levels are synchronized with changes in central OXY levels (Wang et al., 2013; Carson et al., 2015; Baracz et al., 2016). Besides other effects, this hormone regulates social relationships and sexual bonding, as well as aggression, stress, and anxiety (Baskerville and Douglas, 2010).

Methamphetamine (MA) is a highly addictive psychomotor stimulant that is often abused by drug-addicted women during pregnancy (Marwick, 2000; Šlamberová, 2012). A possible explanation for the preference may lie in the effects of MA relative to increased energy, euphoria, and suppressed appetite (Kish, 2008). Additionally, the development of affected fetuses may be impaired since MA easily crosses the placental barrier (Dattel, 1990). Our previous studies have shown that prenatal MA is associated with serious cognitive and psychosocial problems that can persist until adulthood (Šlamberová, 2012; Macúchová et al., 2017). These observations are in agreement with other clinical studies providing evidence of affected children having difficulties in learning and memory, behavioral problems, and intellectual impairment (Baracz and Cornish, 2016). In clinical studies, MA increased maternal depressive symptoms and parenting stress (Liles et al., 2012). The offspring are therefore often neglected and exposed to neonatal stressors (Larsson et al., 1979; Šlamberová, 2012). Moreover, preclinical studies have found that maternal injections during pregnancy, regardless of injected substance (drug or saline), induce long-term impairment of stress responsiveness in adult offspring (Peters et al., 1986; Šlamberová et al., 2002; Bernášková et al., 2017). Injections may therefore be an important prenatal stressor.

The mitigating effects of OXY on MA-related behaviors have been repeatedly described in several experimental protocols suggesting OXY/MA interaction at various neurotransmitter levels (Baracz and Cornish, 2016). However, the MA modulation of endogenous OXY release in adolescents has not yet been studied. With regard to acute effects, there is one study describing enhanced plasma OXY levels after acute administration of MDMA (ecstasy) to adult male rats (Thompson et al., 2007). In adult male rats, it was shown that long-term i.v. self-administration of MA caused increase of plasma OXY levels and at the same time, downregulation of OXY receptors in nucleus accumbens core (NAc) (Baracz et al., 2016).

**Figure 1:**
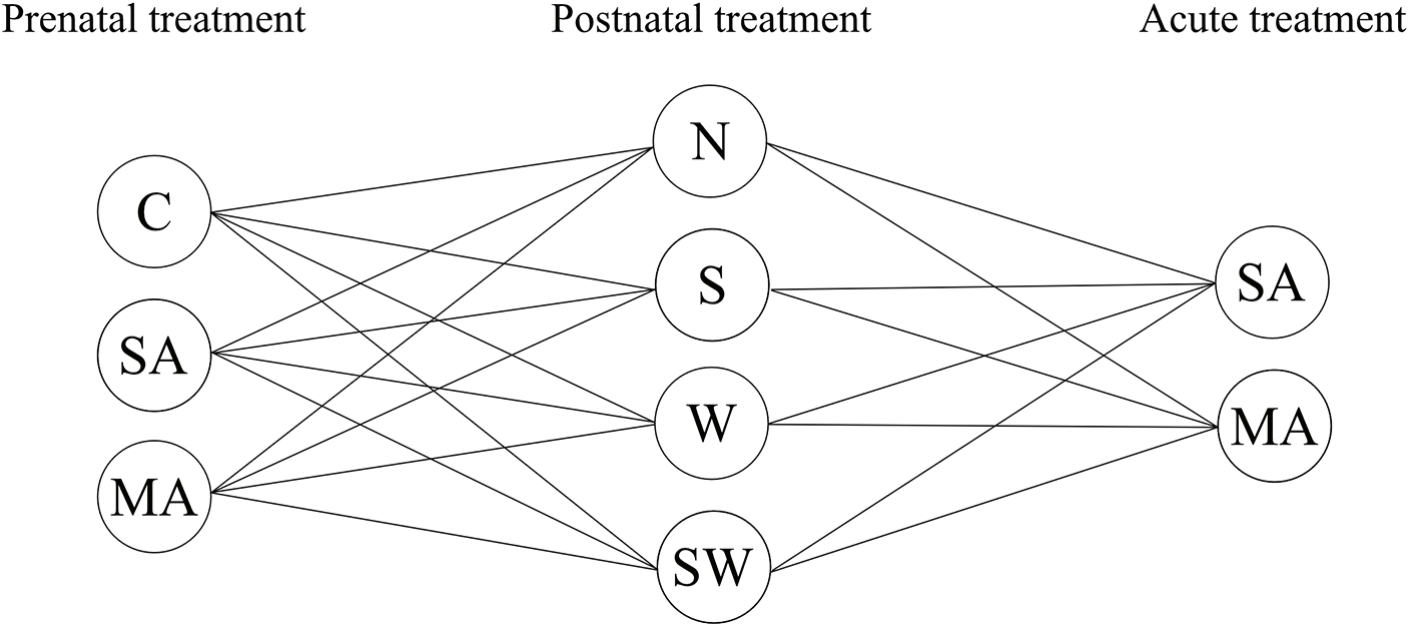
Overview of groups used in present study. Prenatal treatment: C, control; SA, saline; MA, methamphetamine; postnatal treatment: N, non-stressed controls; S, social stress by maternal separation; W, physical stress by maternal cold water; SW, combined social and physical stressor; acute administration 1 h before sample collection: SA, saline; MA, methamphetamine.

The present study used a multifactorial experimental design resulting in 24 treatment groups (Figure 1). We used three groups of prenatal treatment (control, stress, and MA) and four groups of postnatal treatment (control, social stressor, physical stressor, and combination of both). On the final day of the experiment, all groups were divided in two individual groups with single (acute) MA/saline administration. The aim of present study was therefore: (1) to examine OXY release after each perinatal factor used in the present study, i.e., long-term prenatal MA/stress exposure, long-term postnatal maternal stress, and a single MA administration in juvenile female rats; (2) to examine the combined effects of these factors on plasma OXY levels in female progeny at 30 days of age. We chose female adolescent rats because this period is critical for OXY system development (Johnson and Buisman-Pijlman, 2016), and physiological OXY levels in females are inevitably important for later normal maternal behavior, consequently affecting offspring brain development (Toepfer et al., 2017).

## Materials and Methods

The procedures for animal experimentation utilized in this study were reviewed and approved by the Institutional Animal Care and Use Committee and are in agreement with the Czech Government Requirements under the Policy of Humans Care of Laboratory Animals (No. 246/1992) and subsequent regulations of the Ministry of Agriculture of the Czech Republic (No. 311/1997).

### Prenatal Care

Forty eight nulliparous adult female albino Wistar rats (250–300 g) were delivered by Velaz (Prague, Czech Republic) from Charles River Laboratories International, Inc. The dams were housed in groups of four per cage and left undisturbed for 1 week. After 1 week of acclimatization, females were smeared by vaginal lavage to determine the phase of estrous cycle. At the onset of the estrus phase, they were housed overnight with mature males. The females were than smeared again for the presence of sperm and returned to their home cages. The day after impregnation was counted as day 1 of gestation. The females were then randomly assigned to those, who received: (1) MA (s.c., 5 mg/kg), (2) SA (s.c., 1 ml/kg), or (3) no injections (C, controls), during the entire period of gravidity. From the beginning, the rats were housed in a temperature-controlled (22–24°C) colony room, with food and water *ad libitum,* and a 12 h light/dark cycle, with lights on at 6:00 am. On day 20 of gestation, each female was placed into her own maternity cage. The day of delivery was counted as postnatal day (PD) 0.

### Postnatal Care

On PD 1, litter size was adjusted to 12 pups with equal numbers of males and females, whenever possible. The pups were cross-fostered; one mother raised four pups from the MA group, four pups from the SA group, and four pups from control mothers. For recognition, prenatally MA-exposed pups were intradermally injected with India ink in the left hind paw, SA-exposed pups in the right hind paw, and controls were not tattooed. Litters were then divided into four groups relative to exposure to postnatal stress (*n* = 10–12 per group of prenatal treatment): controls (N), maternal separation (S), maternal cold-water stress (W), and maternal separation plus maternal cold-water stress (SW). Stress-exposure was conducted once daily, starting on PD 1, until they were weaned on PD 21.

#### Social Stress

Maternal separation, as a social stressor, was conducted from PD 1–21, for 3 h per day, between 8:00 and 11:00 am (Lajud et al., 2012; Holubová et al., 2016). All pups (from appropriate groups S and SW) were gently removed from their maternity cage and placed in a separate cage in another room. The cage with pups was always placed on a heating pad to maintain normal body temperature.

#### Physical Stress

Maternal cold-water stress was used as a physical stressor for mothers (Drago et al., 1999; Šlamberová et al., 2002). A plastic container (25 cm × 35 cm × 40 cm, LWH) was filled with 5°C water to a depth of 25 cm. Each rat-mother (from appropriate groups W and SW) was placed in the cold water and forced to swim for 5 min. Rats were then towel-dried and placed under a heating lamp until they were mostly dry, and then returned to their home cages. Water in the containers was cleaned of released feces after each animal.

### Plasma OXY Determination

The female progeny at 30 days of age were used to determine plasma OXY levels. The number of offspring used in each group was 10–12. Acute MA or SA (controls) treatment was administrated 1 h prior to blood collection. The reason, why 1 h interval was used in the present study is the finding that the peak of MA levels in the brain is after 1 h after single injection (Rambousek et al., 2014). The rats were sacrificed by decapitation, 2 ml of trunk blood was collected into precooled plastic tubes containing 1,000 KIU (500 KIU/ml) of Aprotinin (SIGMA). After plasma separation in a cooled centrifuge, OXY was immediately extracted according to Szeto et al. (2011). Solid phase extraction was performed on C18Sep-Pak columns, which had been first equilibrated with 3 ml acetonitrile, and then twice washed with 3 ml of 0.1% trifluoroacetic acid. One ml of plasma was mixed with an equal volume of 0.1% trifluoroacetic acid, centrifuged at 14,000 g for 20 min at 4°C. The cleared, acidified plasma was applied to the column. The flow-through was discarded, and the column was washed with 3 ml of 0.1% trifluoroacetic acid and twice with 3 ml of deionized water. OXY was eluted from the column with 3 ml of 60% acetonitrile; the solvent was evaporated under nitrogen. The dried samples were stored at −70°C until assayed. The quantitation of OXY was performed with the use of an Oxytocin ELISA kit from Enzo (cat. No. ADI-900-153A) according to the manufacturer’s instructions. The efficiency of extraction was 88%. The intra- and inter-assay variation was less than 11% and less than 16%, respectively, what meets the criteria given by the manufacturer’s instruction.

### Statistical Analyses

A multifactorial ANOVA (*prenatal treatment × postnatal stress × acute treatment*) was used. The Fisher *post hoc* test was used for comparisons. Differences were considered significant if *p* < 0.05. Data were expressed on graphs as mean ± SEM.

## Results

### The Effect of Prenatal and Acute Treatment

**Figure 2:**
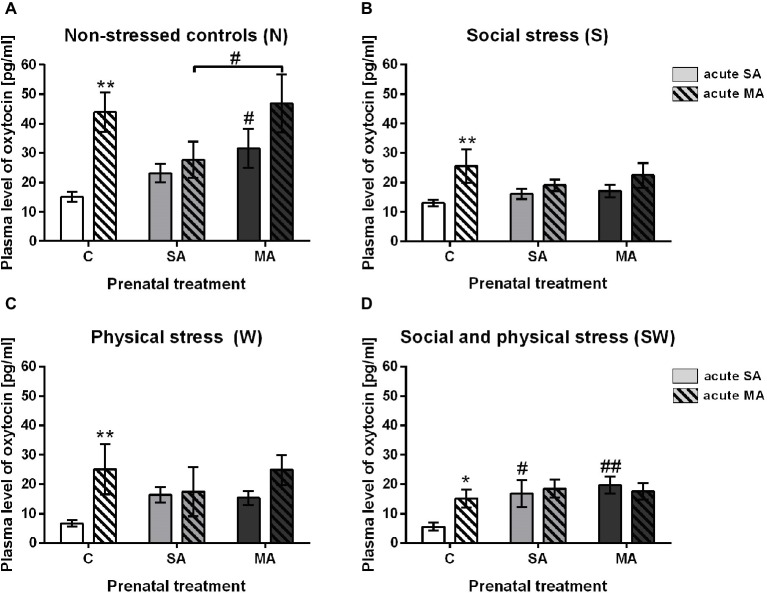
The effect of acute drug administration relative to prenatal treatment. **(A)** Non-stressed controls, **(B)** social stress, **(C)** physical stress, and **(D)** social and physical stress. Values are shown as means ± SEM. C, control; SA, saline; MA, methamphetamine. *p < 0.05 and **p < 0.01 indicate significant differences between acute SA and acute MA administration in appropriate prenatal groups. #*p* < 0.05 and ##*p* < 0.01 indicate significant difference between C and SA or C and MA groups of the appropriate acute treatment.

Plasma OXY levels were found to be significantly increased in the group with acute drug administration 1 h prior to blood collection. This effect was observed only in the prenatally intact groups. Specifically, in non-stressed controls (group N) (*F*_(2, 65)_ = 1.97; *p* < 0.01), in group S affected by social stress (*F*_(2, 62)_ = 1.39; *p* < 0.01), in group W affected by maternal physical stress (*F*_(2, 63)_ = 5.83; *p* < 0.01), and group SW, combined stressors (*F*_(2, 60)_ = 1.73; *p* < 0.05) (Figure 2). There were no significant changes between acute SA and acute MA administration in the rats prenatally exposed to SA or MA in all groups under study (Figures 2A–D).

Other effects were seen among groups of appropriate acute treatment: In the postnatally non-stressed groups N, prenatal MA exposure increased OXY levels compared to intact controls (*F*_(2, 65)_ = 1.97; *p* < 0.05). Also, OXY response to acute MA was significantly enhanced in prenatally MA treated rats compared to prenatally SA treated rats (*F*_(2, 65)_ = 1.97; *p* < 0.05) (Figure 2A). In the SW groups, prenatal SA combined with acute SA showed significantly higher levels of OXY compared to prenatal controls (*F*_(2, 60)_ = 1.73 *p* < 0.05) (Figure 2). The same effect was seen in rats after prenatal MA, and acute SA treatment (*F*_(2, 60)_ = 1.73; *p* < 0.01) (Figure 2D).

### The Effect of Postnatal Stress

**Figure 3:**
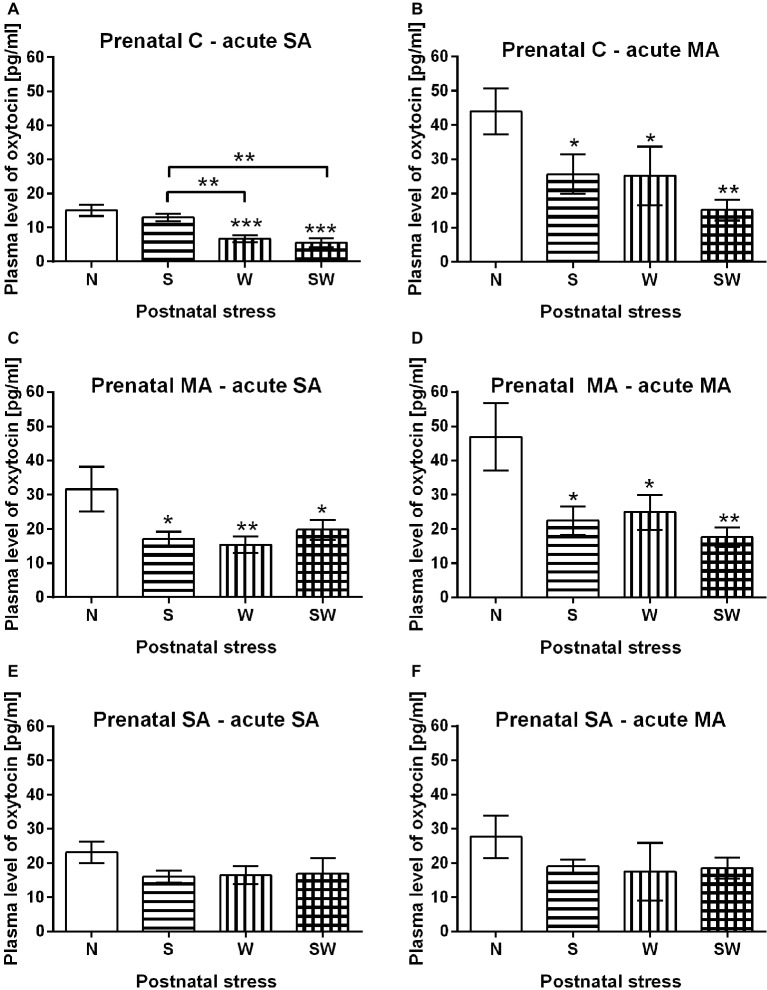
The effect of postnatal stress relative to prenatal and acute treatment. **(A)** Prenatal C – acute SA, **(B)** prenatal C – acute MA, **(C)** prenatal MA – acute SA, **(D)** prenatal MA – acute MA, **(E)** prenatal SA – acute SA, and **(F)** prenatal SA – acute MA. Values are shown as means ± SEM. Prenatal treatment: C, control; SA, saline; MA, methamphetamine; postnatal treatment: N, non-stressed controls; S, social stress by maternal separation; W, physical stress by maternal cold water; SW, combined social and physical stressor. *p < 0.05, **p < 0.01, and ***p < 0.001 indicate significant differences between appropriate groups of postnatal stress exposure.

There was a significant decrease in OXY in postnatally stressed groups compared to non-stressed controls (N) with regard to prenatal or acute treatment. In prenatal controls with acute SA administration (Figure 3A), both groups with physical stress (i.e., W and SW) had significantly decreased OXY levels compared to controls (group N) (*p* < 0.001). This effect was also observed relative to the maternal separation only group (S) (*p* < 0.01), where the OXY levels did not differ from the non-stressed controls (N). Similarly, there was a decrease in plasma OXY levels in all postnatally stressed groups (i.e., S and W) (*p* < 0.05), and SW (*p* < 0.01) after acute administration of MA compared to controls (group N) (Figure 3B). Other similar effect was observed in groups affected by prenatal MA exposure. Postnatal stress groups S and SW (*p* < 0.05) and W (*p* < 0.01) had significantly decreased OXY levels compared to controls (group N) after acute SA administration (Figure 3C) as well as after acute MA administration; groups S and W (*p* < 0.05), and SW (*p* < 0.01) (Figure 3D). However, in prenatally SA-exposed groups different effects became manifested; there were no significant impact of any stress exposure on OXY levels regardless of acute drug administration (Figures 3E,F).

## Discussion

The present study is the first demonstrating the acute MA administration that significantly increases plasma OXY levels in juvenile female rats. This effect was observed in prenatally intact rats only regardless of postnatal treatment. Neither group with prenatal interventions revealed such changes in plasma OXY levels after a single drug administration (Figure 2). Since repeated prenatal MA exposure activated OXY secretion for as long as 23 days, its elevated levels might have downregulated the sensitivity to subsequent single MA administration and no significant additive effect of acute MA was manifested.

Dysregulation of the OXY system has been demonstrated after MA administration in brain regions such as the NAc and the limbic system. Specifically, exposure to MA increased OXY mRNA expression in the NAc in adult male rats (Cadet et al., 2014) and levels of OXY receptors were up-regulated in two regions involved in stress regulation, i.e., the amygdala and hypothalamus in adult male mice (Zanos et al., 2014; Georgiou et al., 2016). Our present data (Figure 2A) on chronic prenatal MA-induced OXY activation in juvenile females are in line with the elevated plasma OXY levels found following chronic MA self-administration in adult male rats (Baracz et al., 2016; Baracz and Cornish, 2016; Georgiou et al., 2016). Similarly, Baracz et al. (2016) showed that increased OXY levels persisted for 15 days after a 20-day period of drug intake. To the best of our knowledge, there are no literary data on gender differences.

It should be noted that OXY has recently been studied as pharmacotherapy for drug dependence. Endogenous as well as exogenous OXY attenuates abuse of various drugs, including MA, *via* modulation of the reward system (Baracz et al., 2016; Baracz and Cornish, 2016). It has been demonstrated, in rodents, that centrally administered OXY reduced MA-induced hyperactivity, acquisition of a conditioned place preference to MA, and relapse-like behaviors, i.e., MA-seeking behaviors (Qi et al., 2009; Cox et al., 2013). Moreover, increased OXY also attenuated self-administration of MA (Carson et al., 2010). The activating MA effect on OXY could contribute to maintain central OXY balance. If we consider that the increased levels of plasma OXY are synchronized with increased central OXY levels (Wang et al., 2013; Carson et al., 2015), then elevated plasma OXY may help restore depletions in the OXY system after drug administration (Baracz and Cornish, 2016).

As mentioned before, acute MA administration significantly increases plasma OXY levels in prenatally intact rats only. Neither group with prenatal stress/drug exposure revealed such changes in plasma OXY levels after a single MA administration (Figure 2). This means that mild prenatal stress of repeated SA injection also plays an important role in the oxytocinergic system development of affected offspring. Results from the present study are supported by those showing elevated peripheral basal OXY levels in the F2 generation after antenatal stress exposure in the F0 and F1 generation (Babb et al., 2014). It has been suggested that chronic elevations of glucocorticoids, after long-term perinatal stress, could impair the hippocampus, amygdala, and prefrontal cortex, all of which have extensive numbers of corticoid receptors (Antonelli et al., 2017). Moreover, our previous study suggested that long-term neonatal stress could lead to subsequent adaptation to stressful stimuli that could manifest as desensitization of adrenocortical corticosterone output in adulthood (Holubová et al., 2016).

Our results show a significant decrease in OXY in postnatally stressed groups. However, this effect was observed only in prenatal controls and the prenatal MA groups (Figures 3A–D). OXY levels in the prenatal SA groups did not vary among the non-stressed and postnatally stressed groups (Figures 3E,F). It supports the observation that mild maternal stress during pregnancy may induce decreased OXY receptor methylation in the offspring, which is associated with increased OXY receptor expression, and may result in greater availability and sensitivity (Unternaehrer et al., 2016). Oxytocinergic system activity, such as this, could dampen the stress response by interacting with the HPA axis and the sympathetic nervous system (Lee et al., 2009; Grewen and Light, 2011). Thus, a fetus would be prepared for a potentially challenging environment through epigenetic adaptation of OXY receptors (Unternaehrer et al., 2016). This may indicate that moderate prenatal stress exposure of repeated injection could lead to better adaptation to more severe stress exposure later in life.

The observed decline of OXY in postnatally stressed groups (Figures 3A–D), could be explained by previously observed reduction in gene expression of hypothalamic OXY in offspring after chronic stress exposure (Murgatroyd and Nephew, 2013). Other preclinical studies observed decreased number of OXY neurons in the hypothalamus of adult rats after neonatal manipulation (Winkelmann-Duarte et al., 2007; Todeschin et al., 2009). These observations could support the diminished plasma OXY observed in our present study. Additionally, a decreased number of OXY-positive magnocellular neurons were found in rats prenatally exposed to repeated restraint stress and altered maternal care during the neonatal period, which further increased anxiety-like behavior and aggressiveness in adulthood. Reduction in the number of OXY-positive neurons was further related to reduce secretion of OXY hormone (de Souza et al., 2013). It is assumed that chronic restraint-induced stress exposure during pregnancy is a severe, more potent stressor than the intradermal injections used in the present study. While the mild stressor leads to adaptation of the system to subsequent stress exposure, the severe stressor has an opposite effect. Our results differ from a previous report by Minhas et al. (2016), where stress led to increased plasma OXY levels in prepubertal females (30 days of ages); however, the study is focused on plasma OXY 30 min, after acute restrain stress, which could explain the opposite effect. The significance of type and intensity of used stressor is obvious also in our present study, where stress of maternal separation alone did not evoke such changes as maternal cold water did.

The present study shows that repeated perinatal stressors affect OXY release in juvenile female rats differently. While early postnatal severe stressors downregulated OXY release, mild early prenatal stress prevented this effect. In prenatally untreated juvenile female rats, acute MA administration stimulated basal plasma OXY levels. Prenatal exposure of rats to mild stressor of daily SA injection prevented postnatal stress-induced OXY inhibition and also the stimulatory effect of acute MA. Since prenatal SA injection inhibited both, stress-induced OXY inhibition and acute MA-induced OXY stimulation, our data point out the modulatory role of prenatal mild stress in OXY response to postnatal stressors or MA treatment. Moreover, there are no other studies focusing on the effect of prenatal drug exposure on plasma OXY levels. More studies are necessary to reveal the exact mechanism involved in both genders. Overall, our data provide evidence that perinatal exposure to stress and drugs significantly alter plasma OXY levels. Since there are few studies focusing on changes in peripheral OXY levels after perinatal stress or drug exposure, more detailed studies into peripheral and central OXY are required. The results of such studies will be valuable resources in the study of adverse effects of perinatal stress on various behavior disorders such as depression, anxiety, schizophrenia, autism, and vulnerability to drug abuse.

## Data Availability

All datasets generated for this study are included in the manuscript and/or the supplementary files.

## Author Contributions

AH as PhD student is responsible for the experimental parts, mainly for perinatal care (treatments) of rats, as well as for the present manuscript. SP has been involved in experimental part and is responsible for determination of plasma oxytocin. JJ has been involved in experimental part as well as in the present manuscript. She is responsible for determination of plasma oxytocin. RŠ is a supervisor of AH and head of the department and the laboratory where this study has been conducted. She has been involved in all parts of the present study.

### Conflict of Interest Statement

The authors declare that the research was conducted in the absence of any commercial or financial relationships that could be construed as a potential conflict of interest.
